# Colon diverticulosis adherent to mesh plug migration after laparoscopic hernia repair: A case study and review of literature

**DOI:** 10.1016/j.ijscr.2021.106403

**Published:** 2021-09-13

**Authors:** Mousa Behbehani, Abrar A. AlAtwan, Abdullah AlHaddad

**Affiliations:** Mubarak AlKabeer Hospital, Jabriya, Hawalli, Kuwait

**Keywords:** Diverticulosis, Mesh hernia repair, Mesh migration, Case report

## Abstract

**Introduction:**

Inguinal hernia repair has evolved from open suture methods to mesh repair which is preformed either open or laparoscopically. Mesh hernia repair has improved the outcome in regards to patient care and recurrence rate but it is also associated with a number of complications. The complications of mesh hernia repair such as deep seated infections, mesh erosion and mesh perforation into nearby viscera has been scarcely reported in literature.

**Case presentation:**

We report a 43 years old male case of diverticulosis adherent to a migrated mesh plug from previous laparoscopic inguinal hernia repair procedure.

**Discussion:**

The choice of mesh material, appropriate suture placement and closure of the peritoneum after mesh repair is very crucial to avoid long term mesh complications.

**Conclusion:**

The aim of this case report is to present a rare complication of mesh erosion with colovesical fistula and abscess formation.

## Highlights

• Inguinal hernia repair is one of the most common procedures performed in surgical practice.
• Mesh hernia repair has improved patient outcomes; regardless it is associated with some complications.
• Mesh migration as a complication should be thought of in patients presenting with acute abdomen.

## 1 Introduction

Elective inguinal hernia repair is one of the most common procedures performed in surgical practice. It is estimated that more than 20 million cases are operated on yearly, worldwide. Seventy five percent of abdominal wall hernias are inguinal, with a 25% lifetime incidence in males and 2% in females [1], [2], [3]. Several repair techniques are available, evolving from open suture repair to mesh repairs, which is either performed open or laparoscopically. The mesh laparoscopic repair is becoming more prevalent nowadays, as it is reported to have substantially less pain in the immediate postoperative period, a faster recovery with an earlier return to normal activity, and reduced surgical site infection when compared with the open technique [4], [5]. Laparoscopic mesh hernia repair is the preferred method for patients with bilateral or recurrent inguinal hernia, but is also performed in some primary cases. Two main laparoscopic hernia repair techniques currently exist, the trans abdominal pre-peritoneal (TAPP) and the total extra-peritoneal (TEP). Even with the introduction of new surgical techniques each with its own advantages and disadvantages it is the introduction of mesh that changed the field lowering the recurrence rate to below 5% [6]. Polyprolene mesh is most commonly used and its complications are being increasingly reported. The work has been reported in line with the SCARE 2020 criteria [7].

## 2 Case presentation

A 43 year old male patient was referred to our hospital for an elective laparoscopic sigmoidectomy due to complicated sigmoid diverticular disease. The patient complained of a 4 month history of mild lower abdominal pain that was predominately localized to the left lower quadrant. The pain was associated with alternating bowel habits and several episodes of bleeding per rectum mixed with stool. The patient also recently noticed onset of dysuria, pneumaturia, and fecaluria. His past surgical history is significant for bilateral laparoscopic hernia repair (TAPP) with mesh placement, which was fixed with tacks 7 years previously and varicocelectomy 5 years ago. He has no relevant medical history of note.

**Fig. 1:**
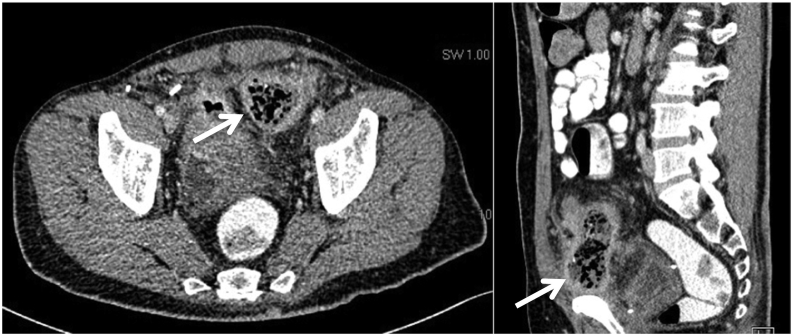
Abdominal computed tomography showed bowel wall thickening and inflammatory stranding involving the sigmoid colon (white arrows).

Examination of the patient revealed mild left lower quadrant tenderness to palpation. All laboratory markers were within normal limits. A CT abdomen and pelvis revealed multiple sigmoid colon diverticula, diffuse mural thickening of mid sigmoid colon with severe adjacent fat stranding, abscess formation adjacent to the sigmoid colon with air pockets sized 3 × 7 × 8 cm (Fig. 1), and a colo-vesical fistula formation causing severe mural thickening in the roof of the urinary bladder with air pockets in the urinary bladder. Colonoscopy revealed multiple sigmoid diverticulitis and inflammatory sigmoid lesion that was biopsied; the lesion however could not be bypassed due to fear of perforation. Biopsy revealed mild chronic colitis and was negative for granuloma or dysplasia.

**Fig. 2:**
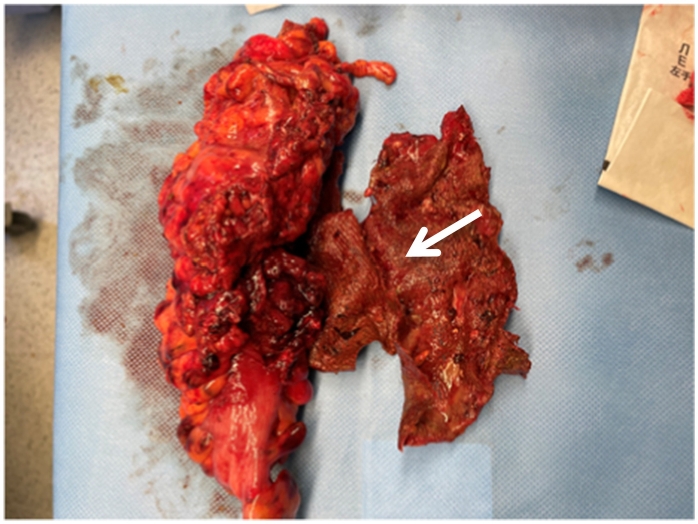
Removal of the mesh plus sigmoidectomy. Arrow, mesh plug.

The patient was admitted and prepped for laparoscopic sigmoid colectomy and excision of colo-vesical fistula. Intraoperative findings revealed a sigmoid colon abscess, a colo-vesical fistula, left inguinal region mesh eroded into the colon from his previous laparoscopic hernia repair (Fig. 2). Abscess drainage, excision of the colo-vesical fistula and sigmoidectomy with removal of the mesh was performed. The right mesh was in place.

Post operatively the patient was started on antibiotics (tazocin), had an abdominal drain that was removed post-operative day 5, and placement of a Foley's catheter for a period of two weeks with a follow up cystogram before removal. The patient had an uneventful post-operative course was tolerating oral meals and discharged on day 6 post operatively. Upon follow up in the outpatients department two weeks after discharge the patient was doing well with no complications reported.

## 3 Discussion

### 3.1 Mesh material

Mesh materials are categorized into synthetic or biologic prosthetics. Synthetic prosthetics are commonly used in hernia repair and are the most common type of mesh employed in abdominal hernia repair, such as the polypropylene mesh (PP mesh) or polytetrafluoroethylene mesh (PTFE mesh) [8]. Synthetic prosthetics can be permeant or absorbable, absorbable mesh can be employed in hernia repair of contaminated wounds, decreasing post-operative infection rates and hernia recurrence [9], [10], while permanent synthetic meshes are contraindicated to be used in a contaminated field [9]. Biologics tend to be used more often in contaminated hernia wounds to assist in wound healing [9], but tend to lead to higher rates of hernia recurrence when used.

Various studies have noted that PP mesh material tends to develop more adhesions over a longer period when compared to PTFE meshes, due to advantages of the microporous nature of PTFE when compared to PP mesh [11], [12], [13]. In a prospective study done in rabbits it was noted that PTFE mesh results in increased shrinkage [14]. It's hypothesized that formation of adhesions can lead to erosion into intra-abdominal organs, while shrinkage encourages mesh migration [11], [14], both of which can lead to devastating complications post mesh placement.

### 3.2 Mesh fixation

The importance of good mesh fixation leads to better wound healing in the early post-operative phase, better mesh integration, and limits mesh mobility reducing the risk of mesh migration [15]. Mesh fixation in surgical practice is most commonly achieved through the use of suturing material or the use of an endoscopic tacker [15], [16]. Sutured groups tend to have more adhesions, increased infection rates, and longer operative times when compared to fixation by use of endoscopic tacker, while not providing superior tensile strength or fixation [16], [17]. The use of a tacker for mesh fixation can lead to nerve and vessel entrapment, which leads to early post-operative pain when compared to groups that undergo suture fixation, but similar levels of pain at 6 weeks post-operatively [15]. The use of a tacker is generally considered a better option for surgeons in laparoscopic hernia repair, the main advantage being the speed of use of a tacker, while a main disadvantage is the cost of tackers when compared to suture fixation [14], [15].

### 3.3 Mesh complications

There has been a growing incidence of mesh related complications in literature, due to the increased use of mesh in laparoscopic and open hernia repairs [19]. The most devastating of these complications are mesh migration and erosion [19], [20].

Mesh migration and erosion are uncommon late onset complications reported from mesh placement. This complication seldom occurs and there is scarcity of data on the subject [15]. Mesh migration can be categorized into primary or secondary migration [11]. Primary or mechanical migration is when a mesh moves along the path of minimal resistance through the anatomical planes, which can be prevented with adequate fixation [11], [14], [22]. Secondary migration tends to be characterized by a gradual trans-anatomic movement that occurs over a longer period of time from erosion of surrounding tissue which is due to foreign body reaction of the mesh material itself.

The main method in which mesh erosion occurs is usually due to the formation of a deep-seated mesh infection which can present months to years after hernia repair. The deep-seated infection forms an enteric or colonic fistula [23], creating an environment of gram-negative bacteria, ultimately leading to mesh failure and neighboring organ involvement.

A reported cause of mesh migration is the presence of a peritoneal defect that allows contact between the mesh and abdominal organs resulting in devastating complications such as bowel obstruction, fistula formation, and adhesions. These complications are more commonly reported with transabdominal preperitoneal (TAPP) inguinal hernia repair than totally extraperitoneal (TEP) inguinal hernia repair, due to the presence of an intraperitoneal dissection and intraperitoneal mesh exposure in TAPP repair [24].

Another theory of mesh migration or erosion is due to the nature of sharp cut edges of the mesh they develop weakening of the wall and erosion of abdominal viscous [24].

### 3.4 Diagnosing mesh migration

The presentation of mesh migration is varied and should be suspected in any patient which has undergone an abdominal hernia repair in the past. Time of presentation can occur soon after surgery or after many years [25]. Patients can present with symptoms of regional abdominal pain or mass formation, urinary or bowel symptoms, cutaneous fistula formation, and fever [26], [27].

To effectively diagnose patients, they should undergo dedicated radiological investigations such as ultrasonography, contrast enhanced CT imaging to assess areas of inflammation abscess formation and presence of any intraabdominal fistula tracts, MRI can be indicated as well to diagnose fistula tracts, cystoscopy can be indicated if patients present with bladder involvement [24]. Colonoscopy can also be employed to assess for any bowel involvement due mesh migration and erosion into the bowel wall which most commonly occur in the small bowel or sigmoid colon [28].

### 3.5 Treatment and prevention of complications

The management of mesh migration depends on the patient's presentation, but it usually involves total excision of the affected mesh [23], [29]. A deep-seated prosthetic mesh infection can initially be treated with drainage and IV antibiotics, but the presence of a deep infection invariably suggests the development of an enteric or colonic fistula that needs to be excised as well.

Erosion into the urinary bladder requires diagnosis via cystoscopy and can be treated conservatively via the use of a Foley's catheter for a 2-week period [20]. The erosion of the mesh in the colon usually requires surgical removal of the involved bowel or colonoscopic removal of the mesh by alligator forceps if complete erosion has occurred [20], [28].

The scarcity of studies that currently exist in literature on the subject of mesh migration or erosion after hernia repair makes it difficult to recommend methods in order to prevent it from happening. Certain objectives to prevent these complications from occurring in which several researches have agreed upon are preparation and placement of a mesh under strict aseptic techniques [29] and proper fixation of the mesh [29], [30]. Direct contact of the mesh with the visceral peritoneum would increase the risk of fistula formation, adhesions, and bowel perforation [31], therefore it is essential for complete peritoneal closure and fixation to be achieved intra-operatively [30], [31].

Factors that impair wound healing can increase the risk of mesh migration and erosion due to inflammatory response, factors such as smoking, diabetes, or other chronic inflammatory conditions, which need to be controlled pre and post operatively.

Polypropylene mesh was the most common material reported in migration cases [11], but it's also the most common type of mesh employed in hernia repair [31]. While biological or absorbable mesh material is not reported to migrate into distant sites, it could be due to the fact these are novels techniques not regularly employed in practice or due to a lack of literature on the subject of mesh migration [11].

Our patient had previously undergone laparoscopic transabdominal preperitoneal bilateral inguinal hernia repair (TAPP repair) with mesh fixation through the use of an endoscopic tacker. His mesh migrated and eroded into the sigmoid colon due to both improper fixation of the mesh and improper closure of the peritoneum. The mesh had eroded into the sigmoid colon and lead to an abscess formation with colo-vesical fistula formation.

This could have been prevented with proper closure of the peritoneum with better fixation either with the use of more endoscopic tackers or the use of both suturing and tackers.

During surgery it is essential to excise the mesh and the inflamed bowel region with anastomosis of the non-disease bowel. At the time of surgery, a biologic mesh can be employed to cover the hernia defect [22] or reimplantation of a new mesh can happen at a later date due to bowel resection contaminating the hernia field [19], [22].

### 3.6 Comparing with other cases present in literature of inguinal hernia repair

A total of 71 cases were mentioned in literature of mesh migration or erosion occurring post laparoscopic or open inguinal hernia repair. A total of 38 cases post open repair and 33 cases post laparoscopic repair.

Regarding open repair mesh erosion or migration most commonly involved the sigmoid colon a total of 17 cases (44%), followed by small bowel at 12 cases (31%), and then the bladder with 7 cases (18%). In regards to laparoscopic hernia repair, mesh erosion or migration the most common organ involved was the bladder at 16 cases (48%), followed by the sigmoid colon at a total of 12 reported cases (36%), lastly the small bowel at a total of 4 cases (12%).

Regarding type of repair involved with mesh erosion or migration, in open hernia repair the most common technique involved was the mesh plug system being reported a total of 22 cases (57%), followed by Lichtenstein hernia tension free repair with 7 cases (18%), other methods involved were the Stoppa method at 3 cases (7%), and the Prolene hernia system and Kugel method at 2 cases each (5%). In regards to laparoscopic repair the most commonly reported laparoscopic technique involved with mesh migration or erosion was TAPP involved in a total of 21 cases (63%), followed by TEP repair in a total of 8 cases (24%), the rest of the cases did not disclose which method of repair was employed.

The mean time to presentation with mesh migration post open hernia repair was a mean of 7 years till development of symptoms. While in regards to mesh migration post laparoscopic repair it was at a mean of 4 years to development of symptoms.

Symptoms of initial presentation are varied and depend on the organ involved symptoms include, but are not limited to PR bleed, abdominal pain, recurrent UTIs or hematuria, small bowel obstruction or large bowel obstruction, fistula formation entero or colo cutaneous, entero or colo vesical fistula formation.

The most common investigative method was a CT abdomen noted in 20 out of 71 cases (28%) followed colonoscopy 16 out of 71 cases (22%), cystoscopy in 15 out of 71 cases (21%), fistulogram or fistulography for 5 out of 71 (7%), other less common diagnostic methods were plain film abdominal x-rays for presentations of bowel obstruction, ultrasound and MRI. Rest of the cases were either diagnosed intra-operatively or authors did not disclose the method of investigation.

The most common mesh involved was the polypropylene mesh used in 57 out of 71 cases (80%) followed by PTFE mesh 2 out of 71 cases (2%), while11 cases did not report type of mesh used (15%).

All cases involved total excision of the mesh and resection of the effected organ via open or laparoscopic techniques.

In conclusion, complications of mesh hernia repair can be wide and involve any organ in the surrounding area such as the bladder and colon. Therefore the stress of choosing mesh material, appropriate suture placement and closure of the peritoneum after mesh repair is very crucial to avoid long term mesh complications.

## Provenance and peer review

Not commissioned, externally peer-reviewed.

## Consent

Written informed consent was obtained from the patient for publication of this case report and accompanying images. A copy of the written consent is available for review by the Editor-in-Chief of this journal on request.

## Ethical approval

N/A.

## Funding

N/A.

## Guarantor

Abrar A AlAtwan.

## Research registration number

N/A.

## CRediT authorship contribution statement

Mousa Behbehani: Conceptualization, Methodology, Writing - Original draft, Writing - Review & editing
Abrar A AlAtwan: Conceptualization, Methodology, Writing - Original draft, Writing - Review & editing
Abdullah AlHaddad: Conceptualization, Methodology, Writing - Original draft, Writing - Review & editing, Supervision.

## Declaration of competing interest

N/A.
